# Simplified flow cytometry-based assay for rapid multi-cytokine profiling and machine-learning-assisted diagnosis of inflammatory diseases

**DOI:** 10.3389/fphar.2025.1594141

**Published:** 2025-06-27

**Authors:** Qiang Quan, Xuegui Ju, Guangmei Li, Lu Ye, Sichong Ren, Shuxin Yang, Rui Zhang, Hui Wang, Ruyue Lin, Luoting Yu

**Affiliations:** ^1^ Department of Biotherapy, Cancer Center and State Key Laboratory of Biotherapy, West China Hospital, Children’s Medicine Key Laboratory of Sichuan Province, Sichuan University, Sichuan, China; ^2^ The First Affiliated Hospital of Chengdu Medical College, Chengdu, China; ^3^ Department of Clinical Laboratory of The General Hospital of Western Theater Command, Chengdu, China

**Keywords:** cytokines, flow cytometry, one-step, lyophilization, machine learning, inflammatory diseases

## Abstract

**Introduction:**

Multiple cytokines detection represents a more robust way to predict the disease progression than a single cytokine, and flow cytometry (FCM)-based assays are increasingly used worldwide for multiple cytokines profile.

**Methods:**

Inspired by One-step concept of ELISA technology, here we reported the development of one-step FCM-based 12-plex cytokine assay to reduce operation and reaction times, in which all the reagents (including capture-antibody-modified beads and phycoerythrin-labeled detection antibodies) had mixed in the same reaction system and achieved similar performance to the conventional approach. Moreover, we used the lyophilization technique to remove the need for cold storage of reagents to further simplify the assay procedure.

**Results:**

We leveraged our technology to test clinical serum samples from patients with COVID-19 or HBV infectious diseases, and established supervised or unsupervised machine learning models to predict the severity or viral load and get deeper insights into the diseases.

**Discussion:**

Together, our results demonstrate a general and framework for convenient analysis of cytokine panel and have the potential to influence medical research and application in this field.

## Introduction

Analyzing a single cytokine is insufficient to determine the outcome of a complex disease such as COVID-19, and strategies incorporating multiple cytokine profile represent a more robust alternative to predict the patients’ outcome and generate deeper insight (Castro-Castro et al., 2022). A growing number of studies are using multiple cytokine combinations to study diseases, including COVID-19 (Patterson et al., 2021), coronary artery disease (Liu et al., 2022), cancer (Shaw et al., 2014), and Alzheimer’s Disease (Aksnes et al., 2021). These studies proceeded smoothly thanks to advances in technologies to detect and quantify multiple cytokines simultaneously in a single assay, especially Luminex-based or flow cytometry (FCM)-based assays. The Luminex system uses hundreds of microsphere or bead sets labeled with two fluorophores in different ratios to encode different cytokines and allows accurate and fast detection, however, the dedicated requirement for analysis instruments and the upfront costs may restrict the utility and availability of Luminex (Liu et al., 2021). In contrast, FCM is pervasive in clinical and research institutions, and increasing cytokine detection kits based on the principle of FCM are available on the market worldwide (Tang et al., 2024).

**FIGURE 1:**
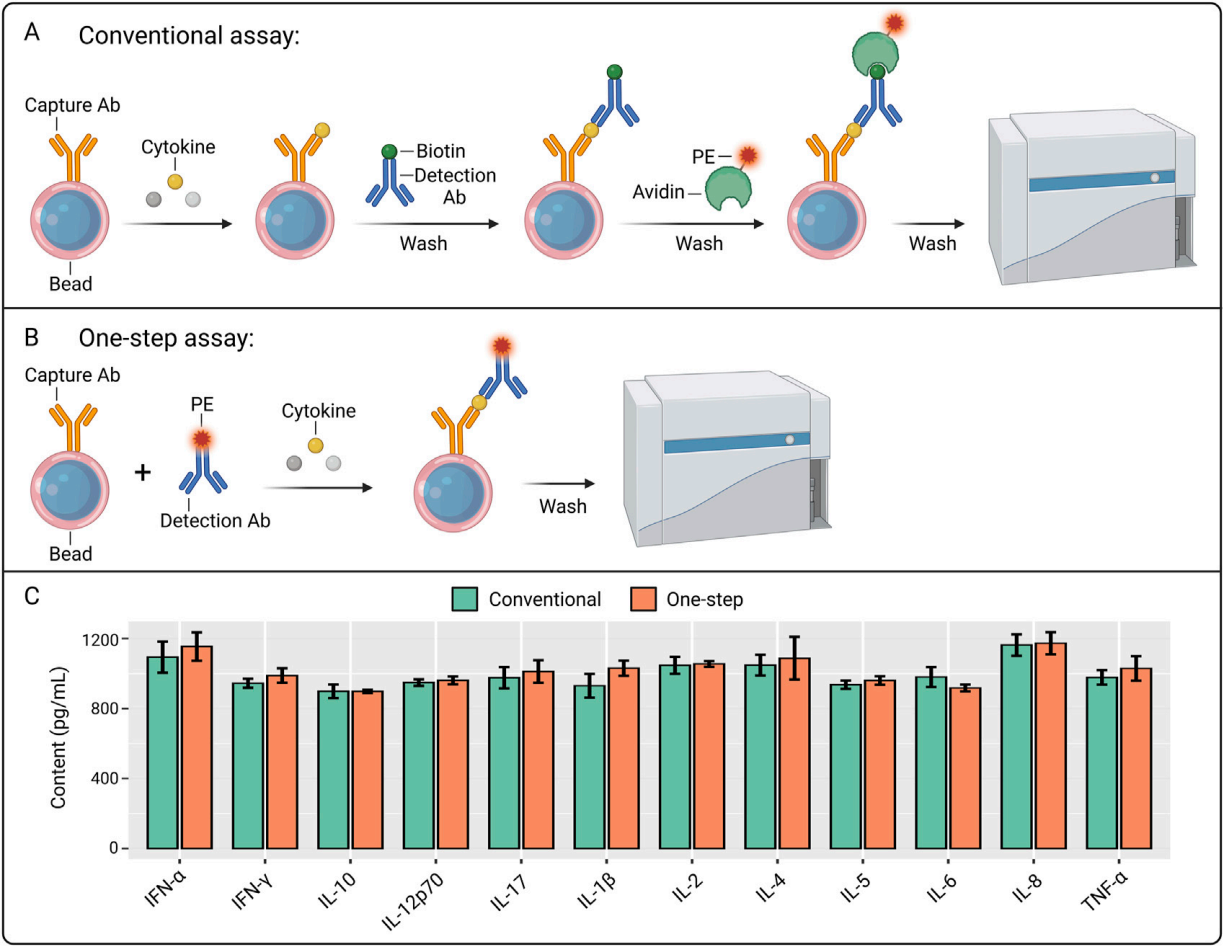
Development of one-step FCM-based multiple cytokine assay. **(A)** Workflow of the conventional FCM-based multiple cytokine assay. The figure was created with BioRender.com. **(B)** Workflow of the One-step FCM-based multiple cytokine assay. The figure was created with BioRender.com. **(C)** The comparison of the detection of twelve cytokines between conventional and One-step approaches.

Here, we lyophilized the assay reagents and achieved a single-step FCM-based detection, therefore effecitvely simplify the reagent transport, storage, and detection procedure (Figure 1). With it, we explored 12-plex cytokine profile in COVID-19 and HBV patients, used supervised and unsupervised machine learning approaches to predict the severity and get deeper insight into the diseases.

## Materials and methods

### Patients

Whole blood was collected in a 5 mL EDTA tube and a 5 mL serum collection tube. A total of 364 individuals were enrolled in the study, including 132 COVID-19 infected patients, 149 chronic HBV infected patients, and 83 healthy individuals. COVID-19 infected patients were defined by a positive result of RT-PCR assay (*i.e.*, Ct value <35 for both *ORF* and *N* genes) of a specimen collected on a nasal or throat swab specimens. The patients were categorized according to the following criteria: Moderate, 1) radiological findings of pneumonia fever and respiratory symptoms, 2) saturation of oxygen (SpO2) ≥ 94% on room air at sea level; Severe, 1) saturation of oxygen (SpO2) < 94% on room air at sea level, 2) arterial partial pressure of oxygen (PaO2)/fraction of inspired oxygen (FiO2) < 300mmHG, 3) lung infiltrate >50% within 24–48 h, 4) heart rate ≥125 bpm, 5) respiratory rate ≥30 breaths per minute. HBV infected patients were positive for both HBV RNA and HBV IgM/IgG serology. Healthy control individuals were recruited from surrounding communities during the same period according to the following inclusion and exclusion criteria:1) age 18–55 years old; 2) not genetically related to the patient; 3) no history of serious physical illness; 4) No history of psychiatric disorders or family history thereof; 5) non-pregnant or lactating women.

### Immune cell flow analysis

1) Add 100 µL whole blood sample in tube incubated with 5 µL each fluorescent antibody (T/B/NK: CD45-PerCP/CD3-FITC/CD4-PE-Cy7/CD8-APC-Cy7/CD16-PE/CD56-PE/CD19-APC; Treg:CD45-PerCP/CD3-FITC/CD4-PE-Cy7/CD25-PE/CD127-APC) for 30 min 2) Add 2 mL of hemolysin, mix in a gentle swirl, and incubate for 10 min with light closed. 3) Centrifuge 300 *g* for 5 min, discard supernatant. 4) Add 2 mL PBS to vortex mix, centrifuge 300 *g* for 5 min, discard supernatant. 5) Add 500 µL PBS vortex again and mix well. 6) The CD4 T cells, CD8 T cells, NK cells, Tregs cell number were tested by the Beckman Coulter DxFlex.

### Measurement of ALT and AST

Serum levels of alanine aminotransferase (ALT) and aspartate aminotransferase (AST) were measured using detection kits from Beijing Strong Biotechnologies, Inc. on a Beckman Coulter AU5800 fully automated biochemical analyzer.

### Quantification of HBV DNA

Serum Hepatitis B virus DNA levels were quantified using the Real-Time PCR Nucleic Acid Detection Kit (Fluorescent Probe Method) manufactured by Sansure Biotech Inc. Extraction of DNA from serum samples was performed following the manufacturer’s protocol, and the viral DNA was amplified and detected on an Applied Biosystems 7,300 Real-Time PCR System.

### Multiplex cytokine quantification with conventional or one-step assay

The recombinant protein standards used in this study were procured from BioLegend. Capture antibodies and detection antibodies were procured from BioLegend, BD, or Thermo Fisher Scientific. Carboxylated fluorescently encoded microspheres were obtained from Spherotech. Capture antibodies were conjugated to the fluorescently encoded microspheres using the EDC (1-ethyl-3-(3-dimethylaminopropyl)carbodiimide) and sulfo-NHS (N-hydroxysulfosuccinimide) coupling method. Phycoerythrin (PE) was conjugated to detection antibodies via succinimidyl-4-(N-maleimidomethyl)cyclohexane-1-carboxylate (SMCC) and dithiothreitol (DTT)-mediated crosslinking.

The conventional FCM-based multiple cytokine assay was performed as follows: 1) 50 μL of sample or standard was combined with 50 μL of capture microspheres in a 96-well plate and incubated with shaking at room temperature for 2 h; 2) unbound material was removed by washing buffer (0.15 M PBS, 0.05% tween-20, PH 7.4), followed by the addition of a biotinylated secondary antibody and incubation for 30 min; 3) after further washing to remove unconjugated secondary antibody, streptavidin-PE conjugate was added and incubated with shaking at room temperature for 30 min; 4) following a final wash step, reading buffer (0.15 M PBS, PH 7.4) was added, and the plate was detected using a Beckman Coulter DxFlex flow cytometer.

The one-step assay was performed as follows: 1) 100 μL of sample or standard was added to a 96-well plate containing lyophilized reagent beads and incubated with shaking at room temperature for 1.5 h; 2) following washing to remove unbound components, reading buffer was added, and the plate was analyzed using the Beckman Coulter DxFlex flow cytometer.

### Lyophilization optimization for one-step assay

Three lyophilization buffer formulations were composed as follows: (1) 0.15 M PBS buffer (pH 7.4) containing 0.1% BSA; (2) 0.15 M PBS buffer (pH 7.4) supplemented with 0.1% BSA and 5% trehalose; (3) 0.15 M PBS buffer (pH 7.4) containing 0.1% BSA, 5% mannitol, and 3% trehalose. The capture-antibody containing microspheres of the twelve cytokines and corresponding detection antibodies were mixed in one of the three lyophilization buffer systems. The number of the microspheres was 100,000 particles/mL, and the concentration of the detection antibodies was 2 μg/mL. Using an automated dispensing system, 250 μL aliquots were rapidly dispensed into liquid nitrogen, where they crystallized into lyophilization reagent beads within approximately 10 s and settled to the bottom. The frozen beads were immediately transferred to a pre-cooled vacuum freeze-dryer and subjected to lyophilization for 12 h to obtain freeze-dried reagent spheres.

### Construction of machine learning models

Three supervised machine learning algorithms, *i.e.*, logistic regression (Logi), random forest (RF), and support vector machine (SVM), were performed using the mlr3verse package (version 0.2.8) in R (version 4.0.1) to predict the HBV viral load. The twelve cytokines, along with the ALT and AST features, were included in the model construction. All algorithms were conducted via random search, coupled with 5-fold cross-validation, to identify the hyperparameter combination with the highest Area Under the Curve (AUC). The performance of these models was compared according to accuracy and AUC. The unsupervised machine learning model (*i.e.*, t-SNE) for clustering COVID-19 patients was conducted with R package Rtsne (version 0.17).

### Statistics

All analyses were performed using R statistical software, and results were visualized with ggplot2 (version 3.4.3) (Ginestet, 2011). Student’s t-tests (two-tailed) were employed to evaluate differences between groups. Statistical significance thresholds were defined as following: p-values <0.05 were considered statistically significant and marked with a single asterisk (*), p-values <0.01 were denoted with two asterisks (**), and p-values <0.001 were labeled with three asterisks (***) t-SNE analysis was conducted with R package Rtsne (version 0.17).

### Ethics statement

The study was approved by the First Affiliated Hospital of Chengdu Medical College (#2021CYFYIRB-SQ-30). All methods were performed in accordance with the relevant guidelines and regulations.

## Results

### Development of one-step FCM-based multiple cytokine assay with lyophilized reagent

Traditionally, indirect labeling through biotin-avidin has been used in FCM-based multiple cytokine assay, and multiple round washing steps and adding operations are required, which complicates the operation and increases the reaction time (3–4 h) (Figure 1A) (Qiu et al., 2014). Inspired by the one-step ELISA technology in which capture and detection antibodies are co-incubated in the same reaction step (Pan et al., 2023), we designed One-step FCM-based multiple cytokine assay in which phycoerythrin (PE) was directly labeled to the detection antibodies and all the reagents (including capture and detection antibodies) are mixed in the same reaction system (Figure 1B). Our results showed that One-step assay achieved a similar level of the twelve cytokine standards compared to the conventional method (Figure 1C).

**FIGURE 2:**
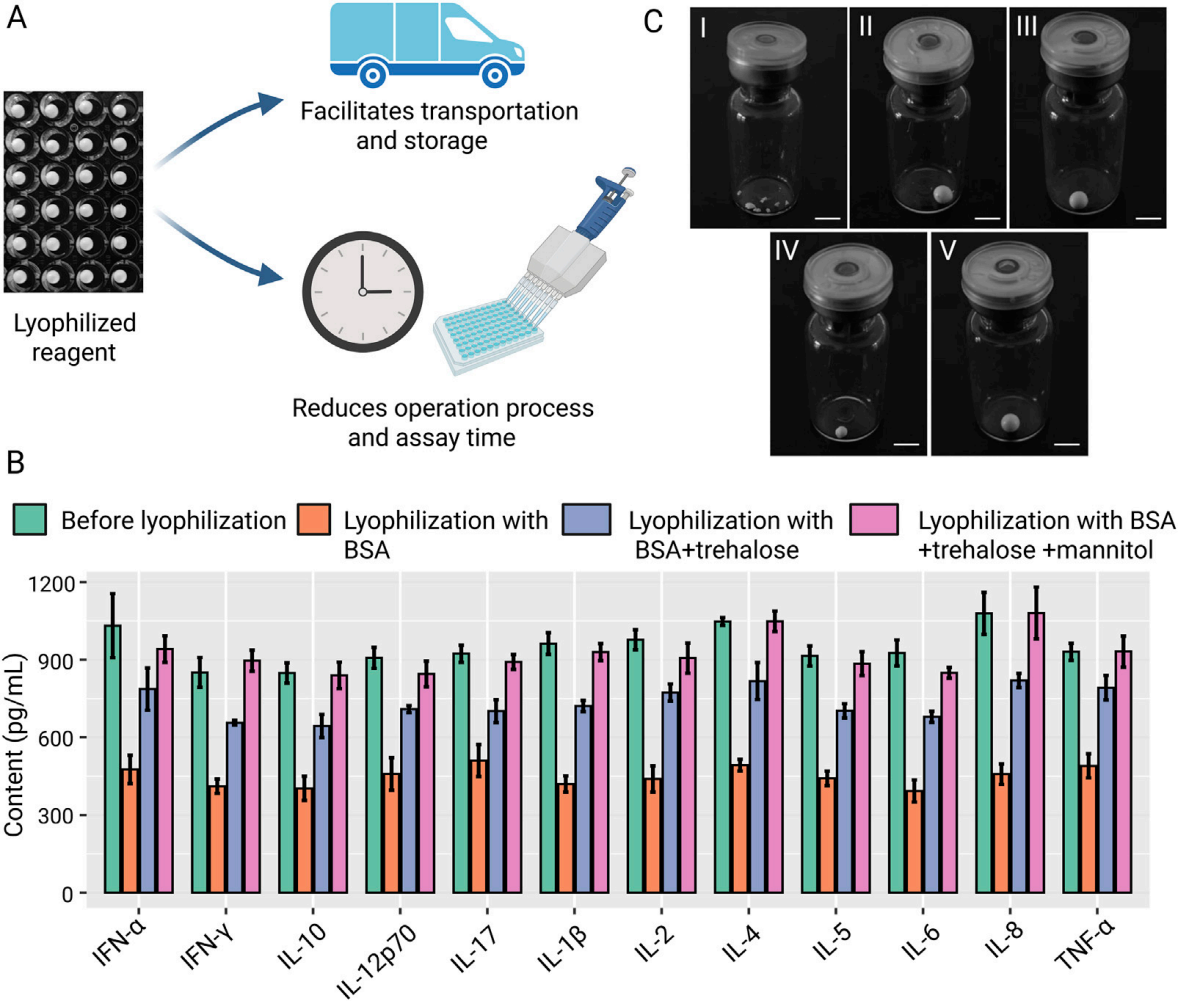
Development of lyophilization technology of the One-step assay. **(A)** Schematic of the advantages of lyophilizing the One-step assay. The figure was created with BioRender.com. **(B)** The performance on the twelve cytokine standards before and after lyophilization using different buffers. **(C)** Representative photo of freeze-dried microspheres: I, lyophilization with 0.1% BSA; II, lyophilization with 0.1 %BSA+5%trehalose; III, lyophilization with 0.1 %BSA+3%trehalose+5% mannitol; IV, 3 days after lyophilization with 0.1 %BSA+5%trehalose; V, 3 months after lyophilization with 0.1 %BSA+3%trehalose+5% mannitol. Bars, 5 mm.

To facilitate the storage and transportation of the reagents and further simplify the assay procedures, we explored the lyophilization technique (Figure 2A). Lyophilizing the initial reaction system (0.1% BSA) substantially reduced the signal for most of the twelve cytokines, compared to the pre-lyophilization reagent (Figure 2B). To remedy this deficiency and obtain freeze-dried reagent spheres, trehalose (5%) or trehalose (3%) + mannitol (5%) were added to One-step reagent as cryoprotectant additives. With combination of the two additives, the One-step assay retained its performance post-lyophilization (Figure 2B). Adding 5% trehalose helped maintain the spherical shape of the lyophilized reagent (Figures 2C–II) while the BSA-only group failed (Figures 2C–I). However, the volume of the reagent sphere decreased significantly after 3 days (Figures 2C–IV), because of absorption of water in the air and subsequent structural collapse (Hammerling et al., 2021; Carpenter et al., 1993; Tonnis et al., 2015). The combination of 3% trehalose and 5% mannitol was conducive for long-term storage (Figures 2C–III), and the shape did not change significantly for 3 months at room temperature (Figures 2C–V).

### A machine learning approach was used to test the new technology on COVID-19 disease and analyze cytokine profile clustering

**FIGURE 3:**
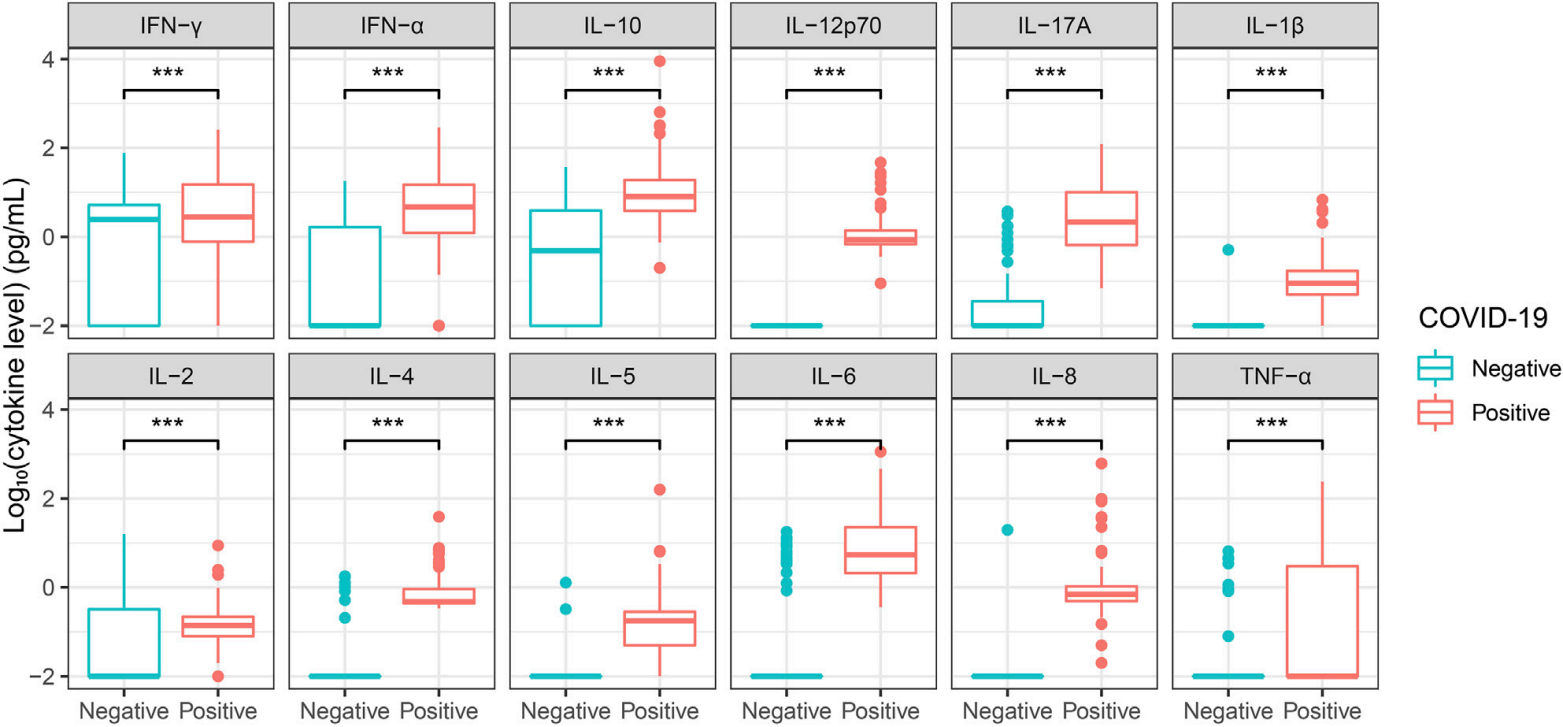
A twelve cytokine panel detection of sera from healthy controls and COVID-19 patients Sera from healthy controls and COVID-19 patients were tested for a twelve cytokine panel. Healthy controls (N = 83) and COVID-19 patients (N = 132). Cytokine concentration was taken the logarithm with base 10 and statistical analysis was performed using unpaired Student’s t-test. ***, P < 0.001.

We tested our one-step FCM-based multiple cytokine assay with clinical serum samples, and selected COVID-19 and HBV infectious diseases. The level of the twelve cytokines in sera samples from healthy controls (N = 83) and COVID-19 patients (N = 132) were assayed (Table 1). All the twelve cytokines were significantly upregulated in the COVID-19 patients compared with that of healthy controls, indicating an antiviral immune response (Figure 3). Consistently, the proportion of the immune cells, such as CD4 cells, CD8 cells, and B cells, were significantly changed upon COVID-19 infection (Supplementary Figure S1). Correlation analysis of patients’ data showed broad relevance within cytokines or immune cell proportions, while a few of negative correlation was observed between certain cytokines and cell proportions, including IL-2, IL-12p70, and IL-6 (Supplementary Figure S2).

**TABLE 1:** Clinical demographic profile. A collection of plasma samples from patients with diagnosed COVID-19 and healthy controls.

Context	COVID-19	Control	*p*-value
N	132	83	
Sex [n (%)] female	71 (53.8)	38 (45.8)	0.316
Sex [n (%)] male	61 (46.2)	45 (54.2)	
Age (mean (SD))	61.2 (21.9)	55.0 (13.8)	0.022

**FIGURE 4:**
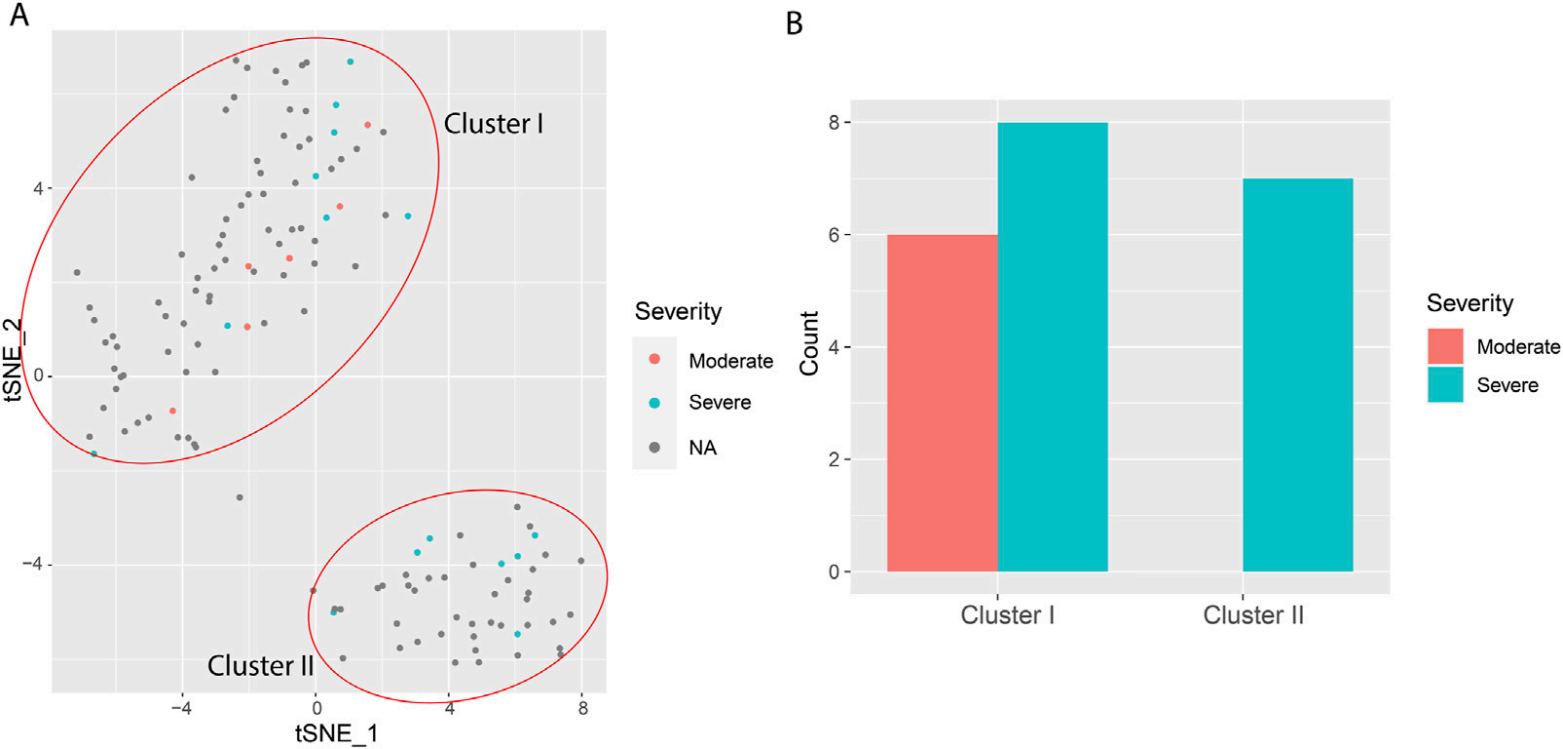
t-Distributed Stochastic Neighbor Embedding (t-SNE) using the twelve cytokine signatures in COVID-19 patients. **(A)** t-SNE map of cytokine analysis of patients. **(B)** Number of available hospitalized patients classified as the two different Clusters.

To probe the potential utility of the twelve-cytokine detection in the disease, we performed t-Distributed Stochastic Neighbor Embedding (t-SNE) with the cytokine data of COVID-19 patients. Intriguingly, the cytokines clustered the patients into two subsets, *i.e*., Cluster I and Cluster II (Figure 4A) (Table 2). All the cytokines, except IL-8, showed significantly higher level in the Cluster II group than that of Cluster I group (Supplementary Figure S3), implying more severe inflammation. The clinical data of 21 hospitalized patients were available, including 6 with moderate symptoms and 15 with severe symptoms (Supplementary Figure S4). Interestingly, all of the six moderate patients were annotated as Cluster I, and Cluster II group only included severe patients (Figure 4B). While the p-value of 0.06 from Fisher’s exact test did not reach statistical significance (Table 2), and future studies with expanded cohorts are necessary to validate predictive utility, our results nonetheless indicate the potential viability of this method.

**TABLE 2:** Baseline conditions for COVID-19 patient clusters.

Context	Cluster I	Cluster II	*p*-value
n	86	46	
Sex [n (%)] female	48 (55.8)	23 (50.0)	0.649
Sex [n (%)] male	38 (44.2)	23 (50.0)	
Age (mean (SD))	61.83 (22.07)	60.07 (21.89)	0.662
Severity [n (%)] moderate	6 (42.9)	0 (0.0)	0.061
Severity [n (%)] severe	8 (57.1)	7 (100.0)	

### Test of the new technology on HBV disease and development of supervised machine learning model

**FIGURE 5:**
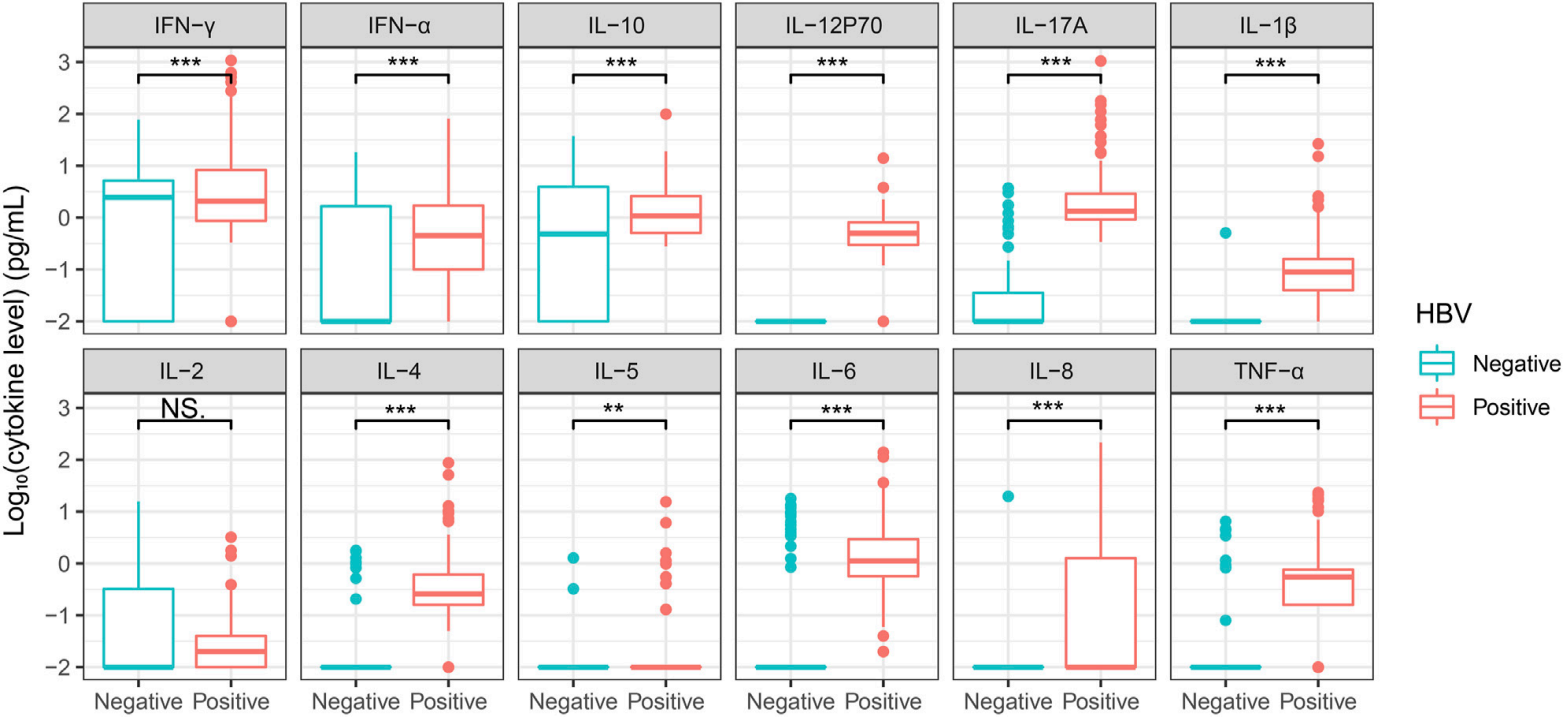
Comparison of the serum level of twelve cytokines in HBV patients and healthy controls Sera from healthy controls and HBV patients were tested for a twelve-cytokine panel. Healthy controls (N = 83) and HBV patients (N = 149). Cytokine concentration was taken the logarithm with base 10 and statistical analysis was performed using unpaired Student’s t-test. NS, Not significant. **, P < 0.01.***, P < 0.001.

**FIGURE 6:**
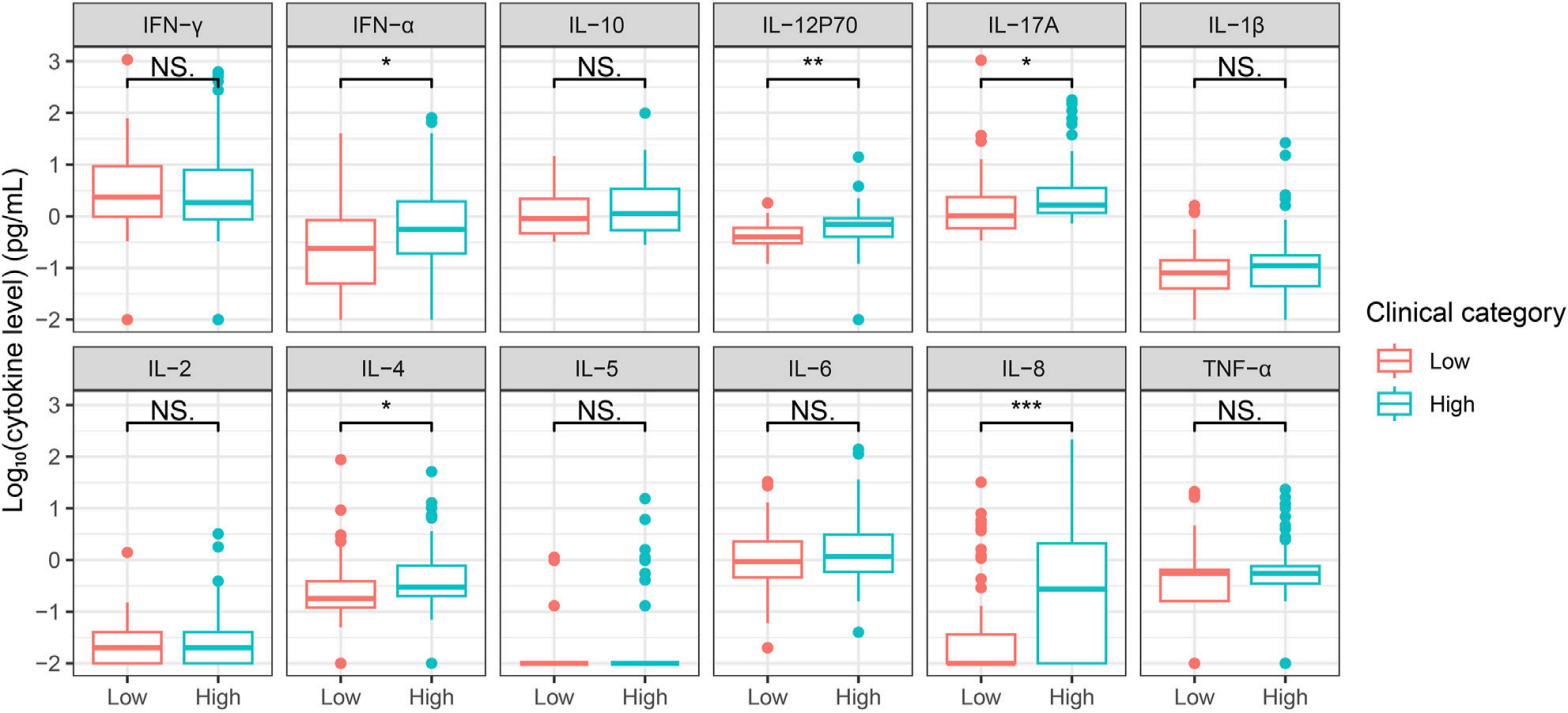
Comparison of the serum level of twelve cytokines in Low and High viral-load HBV patients Sera from HBV patients with low and high viral-load were tested for a twelve-cytokine panel. Low viral load (N = 51), high viral load (N = 98). Cytokine concentration was taken the logarithm with base 10 and statistical analysis was performed using unpaired Student’s t-test. NS, Not significant. *, P < 0.05. **, P < 0.01.***, P < 0.001.

We also detected the cytokine level in sera of patients infected with hepatitis B virus that showed a chronic inflammation (N = 149) (Table 3). All the twelve cytokine levels were significantly upregulated in HBV patients compared with that of healthy control (Figure 5). Monitoring the viral level is crucial for managing the disease, and we divided the patients into low and high viral-load groups based on the value detected by detection kit, in which the value lower than 200 IU/mL was assigned “Low” (N = 51), otherwise “High” (N = 98) (Table 4). Statistical result showed that the content of IFN-α, IL-12 p70, IL-17A, IL-4 and IL-8 in High group were significantly higher than that of Low group (Figure 6), suggesting these cytokines were involved in the more severe inflammation elicited by higher amount of the virus. Levels of serum alanine aminotransferase (ALT) and aspartate aminotransferase (AST) are correlated with the viral load and the disease progression (Supplementary Figure S5) (Shao et al., 2007). Correlation analysis showed that the levels of certain cytokine including IL-6, Il-10, and IL-12p70, were associated with the ALT and AST levels (Supplementary Figure S6).

**TABLE 3:** Clinical demographic profile. A collection of plasma samples from patients with diagnosed HBV and healthy controls.

Context	HBV	Control	*p*-value
n	149	83	
Sex [n (%)] female	74 (49.7)	38 (45.8)	0.667
Sex [n (%)] male	75 (50.3)	45 (54.2)	
Age (mean (SD))	41.8 (12.9)	55.08 (13.8)	<0.001

**TABLE 4:** Baseline conditions for patients with low and high HBV viral load.

Context	Low load	High load	*p*-value
n	51	98	
Sex [n (%)] female	25 (49.0)	49 (50.0)	1.000
Sex [n (%)] male	26 (51.0)	49 (50.0)	
Age (mean (SD))	45.3 (11.2)	39.9 (13.3)	0.015

**FIGURE 7:**
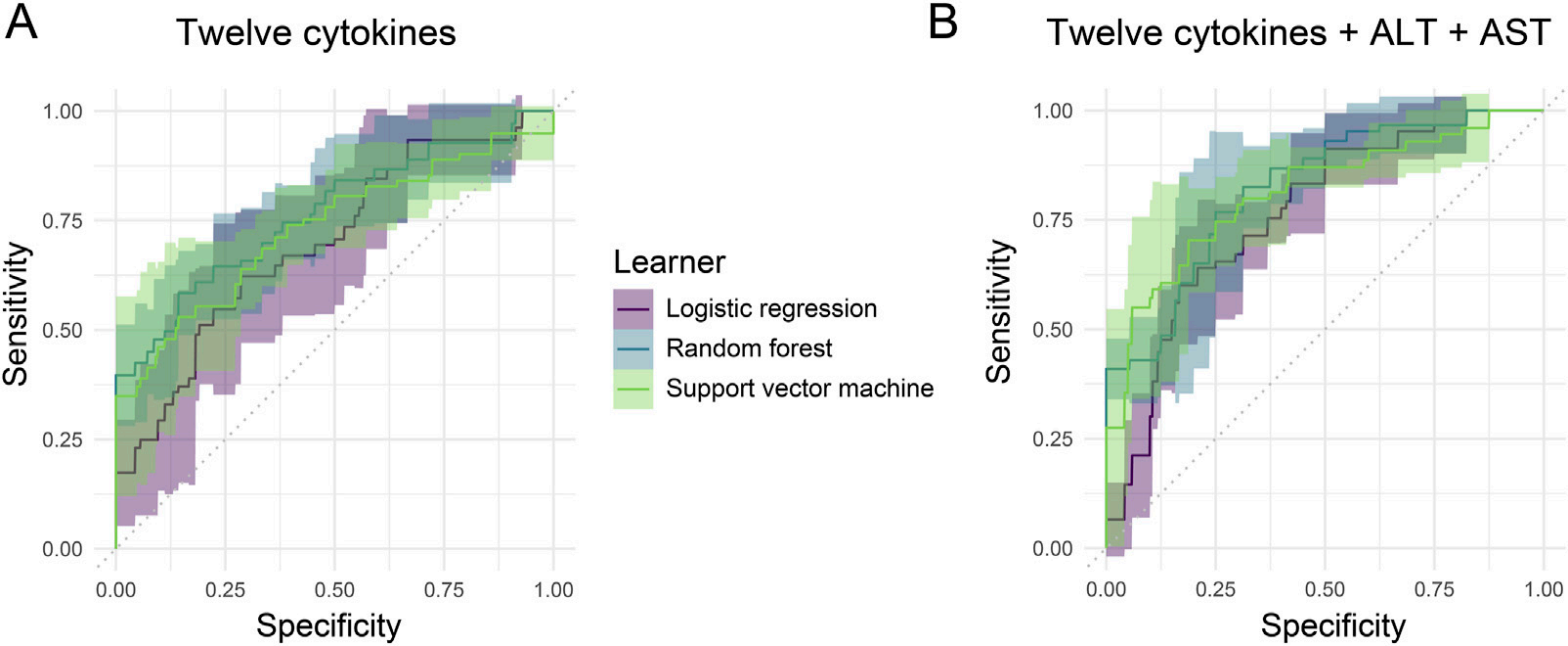
AUROC curves with 95% confidence interval for the machine learning models using a total set of 12 cytokines. **(A)** 12 cytokines only. **(B)** 12 cytokines plus ALT and AST.

Then we investigated whether these twelve cytokine levels in serum could be used to predict the low or high viral load using machine learning (ML). We performed ML modeling with 5-cross fold validation and tested different ML methods, *i.e.*, logistic regression (Logi), random forest (RF), and support vector machine (SVM). Efficient classification of patients based on binary viral levels was demonstrated by the 3 ML models (Figure 7A; Table 5). Respectively, the mean accuracies for Logi, RF, and SVM models were 0.68, 0.76, and 0.72. Moreover, we integrated the ALT and AST features into the ML models and found the performance could be further improved regarding to the AUC score, *i.e.*, reaching to 0.76, 0.85, and 0.82 for the three algorithms (Figure 7B; Table 5). These results supported the utility of the twelve-cytokine detection for the disease evaluation and prediction alone or combination with the classical biomarkers.

**TABLE 5:** Performance of the different machine learning models for HBV level prediction.

Features: Twelve cytokines		
Learner	Accuracy	Area Under Curve (AUC)
Logistic regression	0.68 ± 0.09	0.70 ± 0.10
Random forest	0.76 ± 0.10	0.76 ± 0.09
Support vector machine	0.72 ± 0.08	0.73 ± 0.08
Features: twelve cytokines + ALT + AST		
Learner	Accuracy	Area Under Curve (AUC)
Logistic regression	0.71 ± 0.12	0.76 ± 0.12
Random forest	0.75 ± 0.08	0.85 ± 0.08
Support vector machine	0.76 ± 0.08	0.82 ± 0.08

## Discussion

**FIGURE 8:**
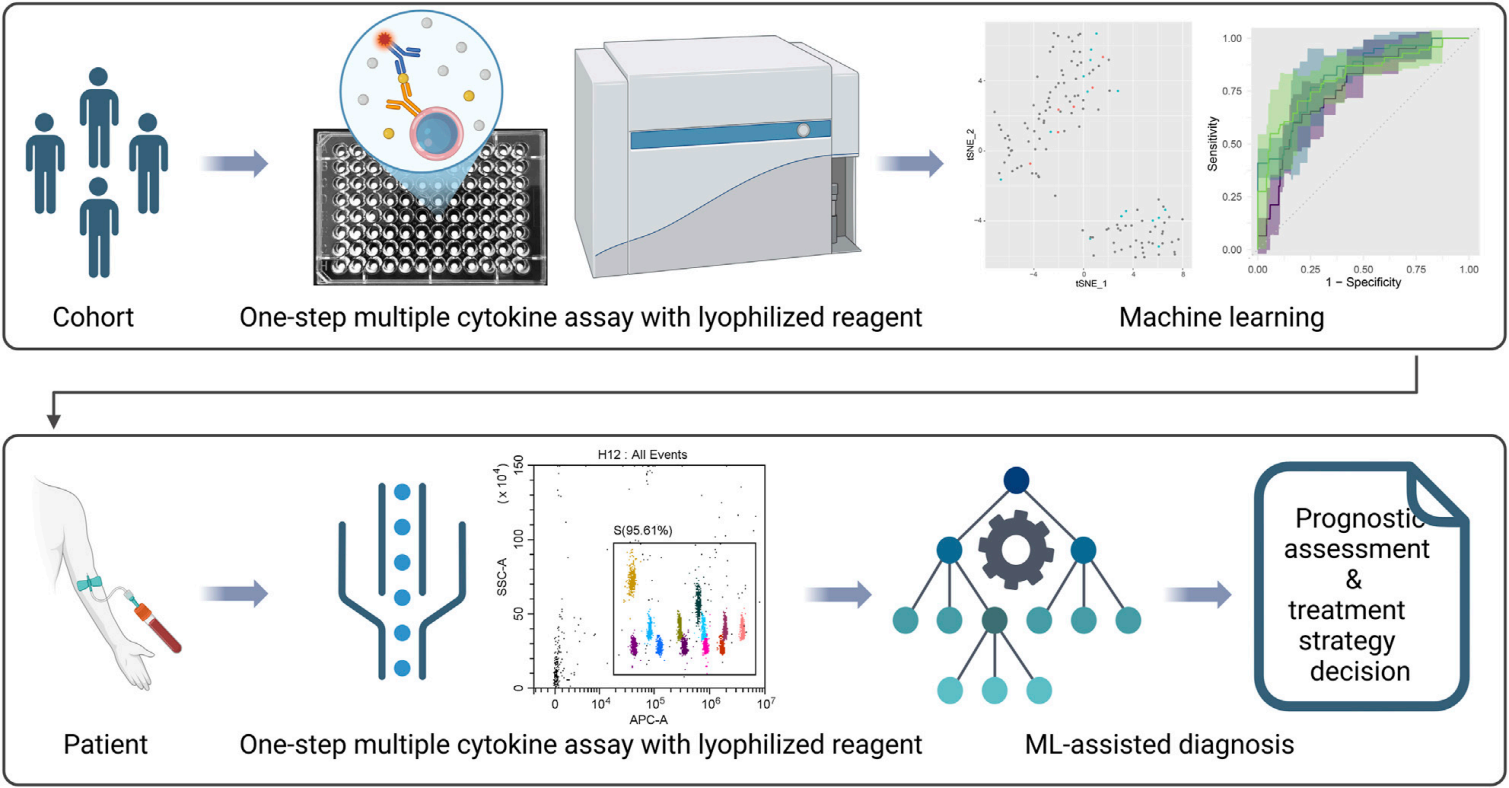
A framework for simplified analysis of cytokine panel and diagnosis of inflammatory diseases. The figure was created with BioRender.com.

Conventional FCM-based multiple cytokine assay uses indirect labeling technology through biotin-avidin system and involves separate storage of individual reagent components, requiring sequential addition during experimental procedures with intermittent washing steps to remove unbound components. While this approach ensures optimal binding at each reaction stage, the complete protocol typically requires 3–4 h for completion (Figure 1A) (Patterson et al., 2021; Djoba Siawaya et al., 2008). In our study, we adopted direct conjugation of PE fluorophore to each detection antibody and optimized formulation, combining capture beads with multiple PE-conjugated detection antibodies in a unified matrix (Figures 1B,C). Moreover, to facilitate the storage and transportation of the reagents and further simplify the assay procedures, we lyophilized the matrix to form a reagent sphere (Figure 2). This integrated reagent sphere undergoes rapid rehydration upon contact with test specimens, simultaneously releasing functional capture beads and fluorescent detection antibodies to form capture bead-antigen-PE antibody complexes. Following a 1.5-h incubation period, a single wash step suffices for removal of unbound sample matrix and excess PE-conjugated detection antibodies prior to instrumental analysis. The complete workflow is completed within 2 h, demonstrating comparable detection efficacy while significantly reducing processing time compared to conventional methodologies. With the developed assay and machine learning technology, we demonstrated a general and framework for simplified analysis of cytokine panel and diagnosis of inflammatory diseases (Figure 8).

Here we chose the specific twelve cytokines, *i.e.*, IFN-α, IFN-γ, IL-1β, IL-2, IL-4, IL-5, IL-6, IL-8, IL-10, IL-12p70, IL-17 and TNF-α. IFNα, IFNγ, IL-12p70, IL1β, IL8, IL6, and TNFα are pro-inflammatory cytokines that have been widely reported in the past (Tang et al., 2024). They are crucial for the inflammatory response and cytokine storm caused by viral infections. IL-2 is an important cytokine for T cell proliferation, IL-17 is a marker of Th17, and IL-4, IL-5, and IL-10 are markers of Th2. These cytokines represent the dynamic changes in Th1, Th2, and Th17 responses. This detection panel can provide a comprehensive response to the body’s innate immunity and T cell immune response. It should be noted that our developed technology can also detect other type and number of cytokines, offering a potential solution to the challenge of high-throughput cytokine assay in clinical settings and therefore providing a comprehensive understanding the immune status of patients.

COVID-19 has created a huge social burden, infecting and killing millions of people worldwide (Pickering et al., 2025). Its progression is marked by dynamic immune responses, with cytokine profiles offering critical insights into pathogenesis and prognosis. While challenges remain in standardization and causality, cytokine-based models hold promise for personalized therapeutic strategies and resource allocation (Zhu and Yao, 2024; Rashidi et al., 2024). For example, an unsupervised machine learning algorithm (Hierarchical Clustering) was used to cluster hospitalized COVID-19 patients in to three categories merely based on 12-plex cytokine panel (IL-1 β, IL-6, IL-8, IL-10, IL-17, TNF, IFN-α, IFN-γ, CXCL10, CCL2, CCL3, G-CSF), and significant differences in mortality rates were found among the clusters (Castro-Castro et al., 2022). Supervised machine learning algorithm (RF) was used to predict the COVID-19 severity and chronicity based on immune subset profiling and a 14-plex cytokine panel (TNF-a, IL-4, IL-13, IL-2, GM-CSF, sCD40L, CCL5, CCL3, IL-6, IL-10, IFN-g, VEGF, IL-8, and CCL4 (Patterson et al., 2021). Whether multiple cytokine panel only can be used to predict the COVID-19 severity other than mortality remains unknown. In this study, we used a 12-plex cytokine panel (IFN-α, IFN-γ, IL-1β, IL-2, IL-4, IL-5, IL-6, IL-8, IL-10, IL-12p70, IL-17 and TNF-α) different from the above, explored an unsupervised machine learning algorithm (*i.e.*, t-SNE), and found patients could be clustered into two groups (Figure 4A). Intriguingly, all of the four moderate patients were annotated as Cluster I, and Cluster II group only included severe patients (Figure 4B). These results suggested multiple cytokine panel alone could predict outcome of COVID-19 patients, and underscored the importance of developing an easy-to-use method for multiple cytokine detection, which might bring new insight about different combination of cytokines in disease progression.

HBV infection causes liver-related morbidity and mortality worldwide. Serum viral biomarkers are crucial for the prognostic assessment and treatment strategy decision in the various clinical guidelines, and serum HBV DNA is the most important biomarker (Mak et al., 2023). Changes in the level of virus in the peripheral blood are associated with the host’s intricate immune response. The correlation between the level of viremia and cytokines in HBV patients is controversial (Zhong et al., 2021; Ribeiro et al., 2022). A recent large-scale meta-correlation analysis of 1,199 HBV patients and several cytokines showed a pooled correlation between the HBV load and cytokines, especially IL-10 and IL-9 (Manea et al., 2024). In this study, we explored the feasibility of establishing a machine learning model using the twelve cytokines to classify the HBV load (High vs. Low) (Figure 7) and compared three classical algorithms (Logi, RF, SVM). The RF algorithm showed the highest accuracy (0.76 ± 0.10) and AUC (0.76 ± 0.09). Together with previous finding (Manea et al., 2024), these results consolidate the correlation of serous level of cytokines with the HBV load and reveal the utility of machine learning model to fit their relationship. Moreover, when integrated with ALT and AST biomarkers, the AUC score of the RF algorithm can be further elevated to 0.85 ± 0.08 (Figure 7; Table 5). It implies that the serous cytokines can be combined with various related biomarkers of different diseases to develop accurate machine learning models to help diagnosis as what has been done in this study.

The twelve cytokine detection method we developed can effectively reduce the clinical testing costs of cytokines. It must be admitted that the selected cytokines do not fully represent the immune status of patients, as there are many other cytokines involved in the immune response against infections and autoimmune diseases, among others (Liu et al., 2021; Schett et al., 2021). Further analysis of the mechanisms of immune dysregulation in different diseases is needed in the future, and a detailed examination of the changes in cytokines should be conducted in order to develop better testing kits to assist in predicting disease progression and prognosis. In this sense, the development of one-step FCM-based multiple cytokine assay with lyophilized reagent will contribute to basic research and clinical translation in this field.

## Data Availability

The raw data supporting the conclusions of this article will be made available by the authors, without undue reservation.
