# Supplementary material for: Simplified flow cytometry-based assay for rapid multi-cytokine profiling and machine-learning-assisted diagnosis of inflammatory diseases
Source: Front Pharmacol. 2025 Jun 27;16:1594141. doi: 10.3389/fphar.2025.1594141 (PMC12245854; doi:10.3389/fphar.2025.1594141)
Supplement: Supplementary file 1 [file Supplementaryfile1.pdf]

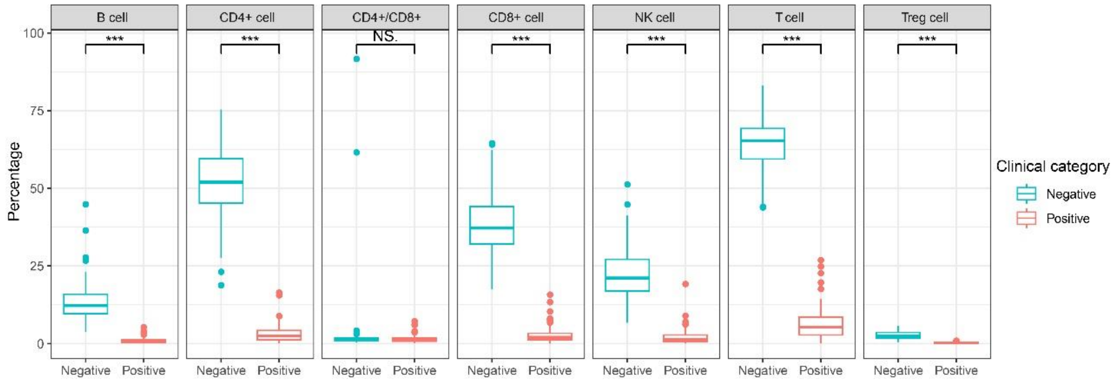

Supplementary Figure 1 Detection of immune cell component proportion in whole blood of health controls and COVID-19 patients

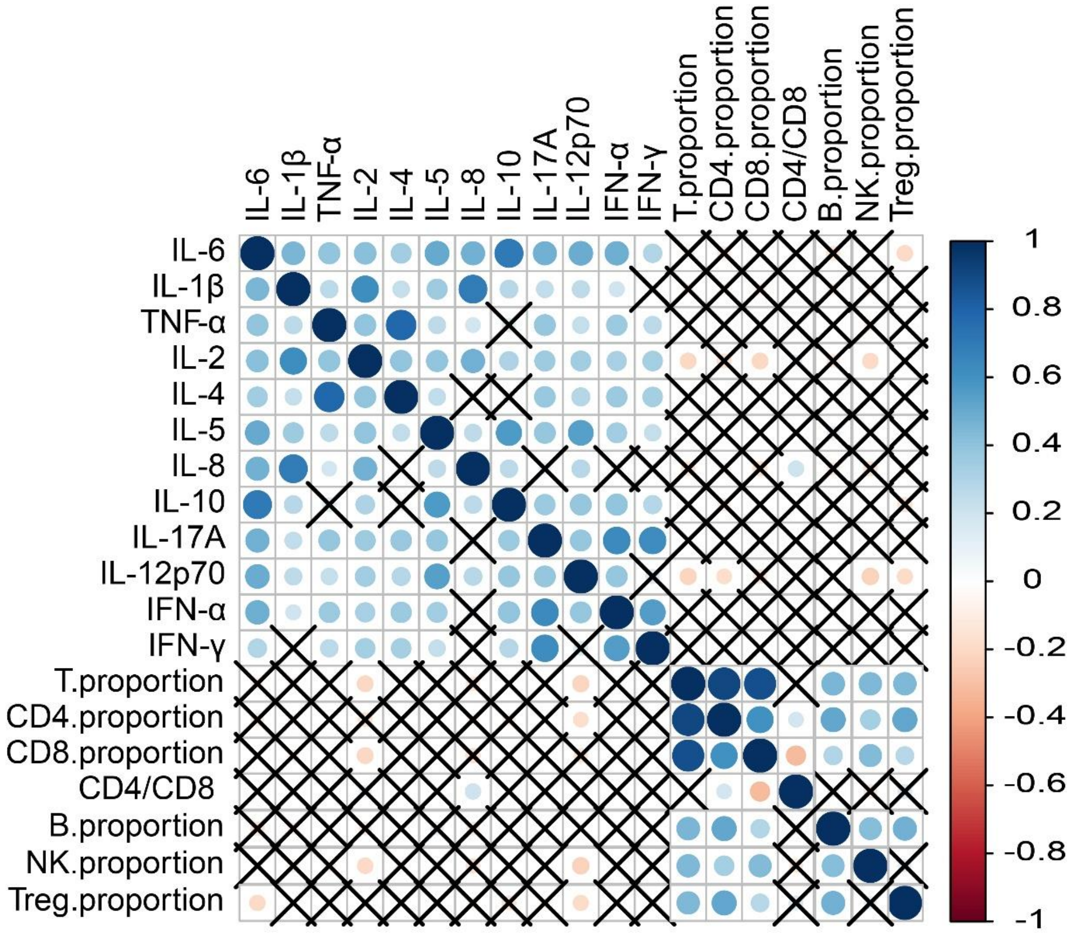

Supplementary Figure 2 Pearson correlation coefficients of cytokine and cell component variables. The X mark indicates that the result is statistically insignificant ($p > 0.05$).

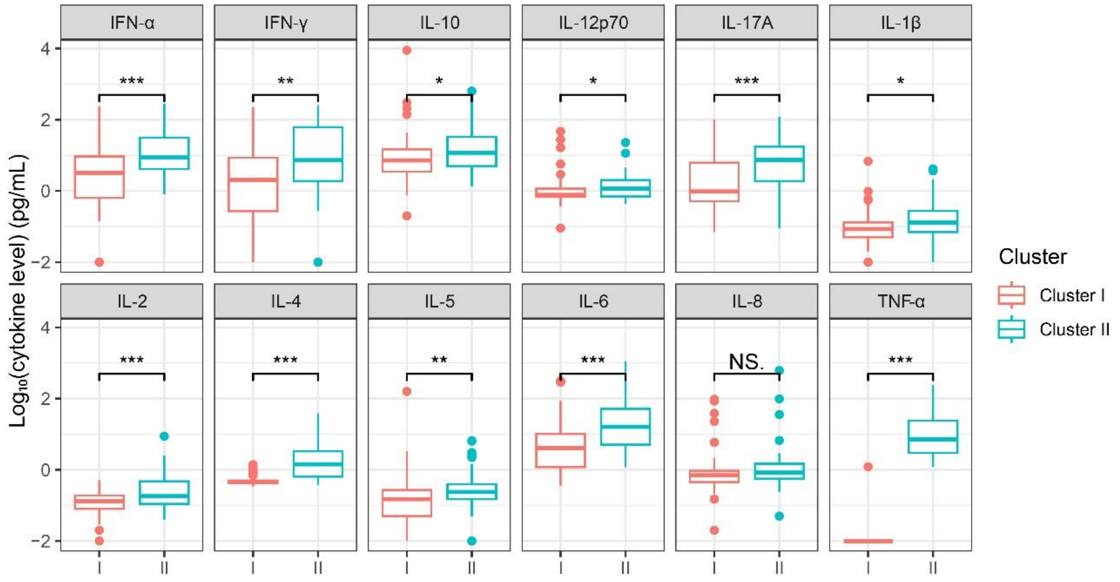

Supplementary Figure 3 Comparison of the serum level of 12 cytokines in Cluster I and II COVID-19 patient groups

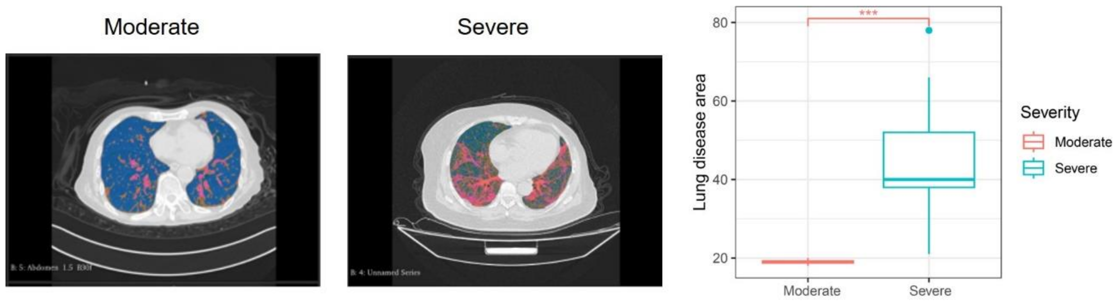

Supplementary Figure 4 Lung disease area in moderate and severe COVID-19 patients and representative images were shown

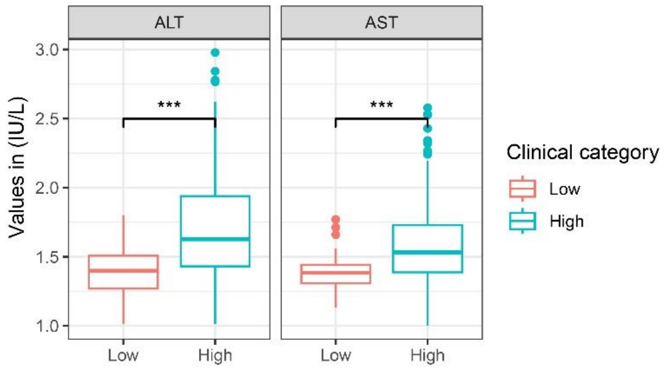

Supplementary Figure 5 Levels of serum alanine aminotransferase (ALT) and aspartate aminotransferase (AST) in subsets of HBV patients

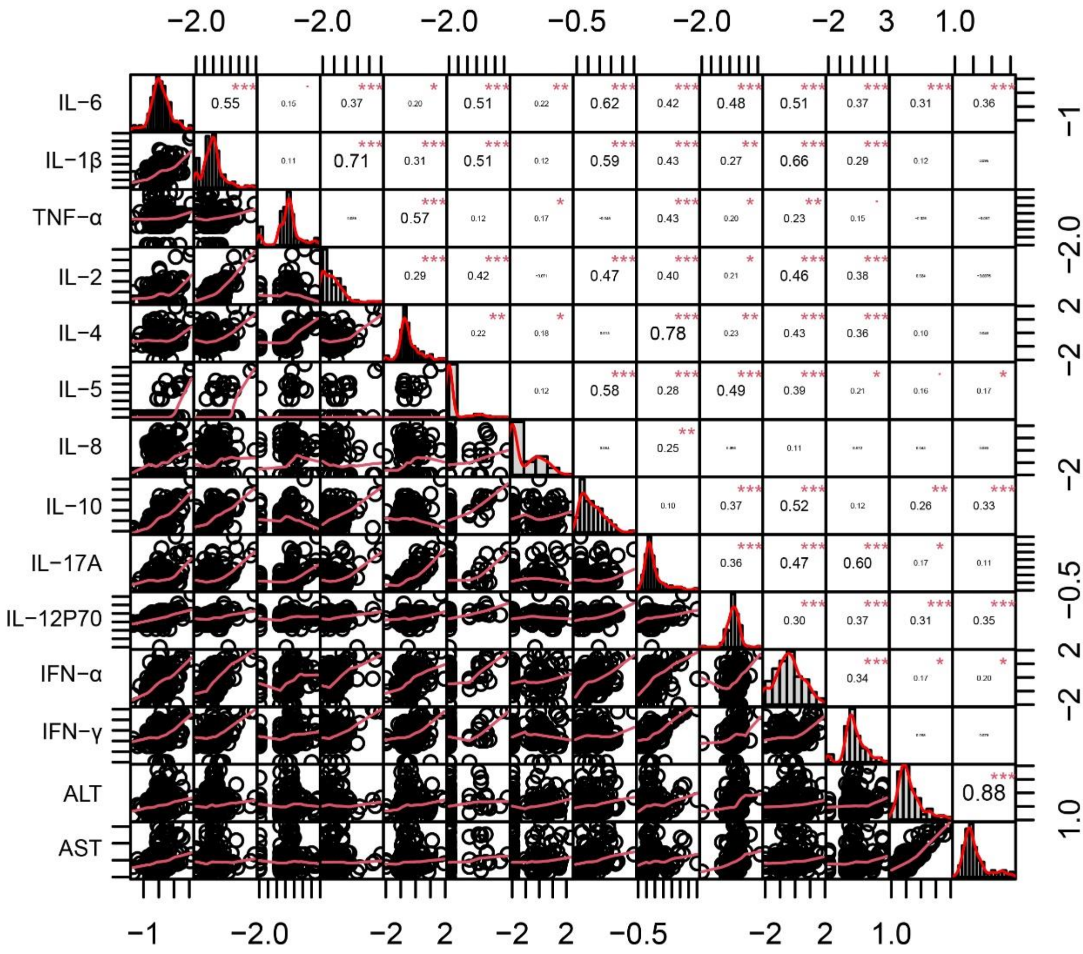

Supplementary Figure 6 Pearson correlation analysis of the twelve cytokines, ALT, and AST in HBV patients
